# Exploring Online Peer Support Groups for Adults Experiencing Long COVID in the United Kingdom: Qualitative Interview Study

**DOI:** 10.2196/37674

**Published:** 2022-05-20

**Authors:** Hannah L S Day

**Affiliations:** 1 Faculty of Public Health and Policy London School of Hygiene and Tropical Medicine London United Kingdom

**Keywords:** COVID-19, long COVID, post-COVID-19 syndrome, peer support, online health communities, self-help groups, internet, qualitative, interview, patient experience, digital health, digital peer support, online health

## Abstract

**Background:**

Long COVID is an emerging public health concern. A growing number of individuals are experiencing prolonged, multifaceted health challenges and accompanying social impacts after COVID-19 infections. Support services in the United Kingdom remain insufficient and fraught with complexity. Responding to persistent gaps in care, patients joined forces in online peer support groups. However, little is known about how these groups impact patients with long COVID and their lived experiences of the condition.

**Objective:**

The aim of this study is to explore the roles that online peer support groups take on and the impact they have on patients experiencing and recovering from long COVID in the United Kingdom. In doing so, this study aims to identify ways to inform future long COVID care, including online peer support and broader long COVID care structures.

**Methods:**

I conducted 11 semistructured interviews virtually on Zoom in July 2021. Participants had long COVID, were UK-based, and used long COVID online peer support groups. Topics discussed in interviews included what led participants to these groups, experiences within them, and feelings about the roles that the groups took on. I analyzed the results by manually conducting thematic analysis.

**Results:**

Long COVID online peer support groups had numerous roles, significantly impacting users. I identified 5 themes and 13 subthemes through thematic analysis. The identified themes were as follows: (1) filling professional care gaps, (2) societal awareness, (3) engagement behavior, (4) diversity, and (5) social connections. Given the void of professional support, those experiencing long COVID gained some benefit from these groups. However, participants emphasized notable concerns about the all-encompassing roles these groups embody and speculated over potential improvements.

**Conclusions:**

If used appropriately, online peer support groups could be immensely beneficial for patient well-being, beyond simply filling gaps in long COVID care. However, it appears many groups take on more than they can manage and become potentially harmful. Through prioritizing patient voices, long COVID care could be restructured to maximize peer support’s benefits within broader care structures.

## Introduction

### Context of Long COVID

#### Prevalence and Impact

The COVID-19 pandemic has significantly impacted the UK population. As of March 29, 2022, there had been 20,986,171 confirmed COVID-19 cases and 164,974 deaths within 28 days of a positive COVID-19 test [1,2]. However, as Tim Spector (creator of the ZOE COVID Symptom Study app) noted in May 2020, there is a misconception that “if you are not dead you are fine,” thus leaving continued suffering unacknowledged [3].

Long COVID encapsulates both “ongoing symptomatic COVID-19” (symptoms experienced for 4-12 weeks from infection) and “post-COVID-19 syndrome” (symptoms 12 or more weeks from infection) [4]. Prevalence estimates in the United Kingdom from a survey completed on March 5, 2022, indicated that 1.7 million people in private households were actively experiencing long COVID symptoms without an alternative explanation [5]. An estimated 784,000 (45%) of those reporting long COVID stated they had COVID-19 over a year prior, and 74,000 (4%) reported it had been at least 2 years since their COVID-19 infection [5]. However, long COVID prevalence estimates vary widely and largely depend on self-reported data [6,7]. A study from February 2021 found that roughly 30% of patients with COVID-19 experienced long-lasting symptoms, even 9 months after infection [8]. This uncertainty is amplified by the widespread unavailability of COVID-19 testing early on. Long COVID may not be considered without confirmed COVID-19 infection [9]. With England’s “Living with COVID-19” plan removing access to universal free COVID-19 tests from April 1, 2022 [10], long COVID’s impacts may be further clouded as many will not have a positive test to document a prior infection.

Long COVID symptoms appear systemic, impacting multiple bodily systems with varying severity [4,11,12]. A survey uncovered 203 long COVID symptoms [11]. Common symptoms include immense fatigue, cognitive dysfunction (“brain fog”), palpitations, peripheral neuropathy, depression, breathing difficulties, autonomic dysfunction, and new-onset allergies [6,11,13]. This incomplete list mirrors long COVID’s all-encompassing nature, unsettling assumptions that long COVID can be simplified into a single unidirectional illness trajectory.

In an Office for National Statistics survey in June 2021, roughly 57% of those self-reporting long COVID reported negative impacts on their well-being and 30% reported negative impacts on work [14]. Compared to those not experiencing long COVID (or any COVID-19 infection), those with long COVID fared worse across numerous indicators, such as anxiety and loneliness [14]. Long COVID does not impact everyone equally [5,6,13]. Women appear twice as likely to experience long COVID than men [15]. The 35-49-year age group was most likely to report long COVID [5], with increased risk for lower-income groups [6]. Preexisting disability and health conditions appear to increase risk [5], particularly existing lung conditions [6]. However, current data should be approached with caution as research is evolving.

#### Definitions and Medicalization

The term “long COVID” was created as a hashtag (#LongCovid) by Elisa Perego in May 2020, naming her turbulent COVID-19 experience [16,17]. This phrase’s popularity grew quickly, shifting into news and research [16]. Notably, there remains uncertainty around long COVID’s definitions. The World Health Organization defines long COVID as symptoms lasting more than 3 months from initial COVID-19 infection [18]. However, the definition from the National Institute for Health and Care Excellence (NICE) used before expands this timeline to symptoms lasting more than 4 weeks [4]. Given that this research focuses on the UK population and corresponding data, I am utilizing NICE’s definition. Additionally, this broader definition avoids unduly excluding patients who require essential services, particularly while long COVID remains poorly understood. Patients had to fight for long COVID recognition, medicalizing the condition. Historically, experts drove medicalization: situating natural experiences within biomedicine’s purview [19,20]. Recently, patients have driven this phenomenon [21], as with long COVID. Long COVID is speculated as being the first illness defined solely through patients’ social media communications [9,16]. Patients achieved validity through “illness reification,” defining suffering through shared experience [21], thus accessing medical legitimization and care.

#### Existing Health Care Response

The National Health Service (NHS) has attempted to provide long COVID support. In the summer of 2020, NHS England launched “Your COVID Recovery,” a website connecting patients with health care providers (HCPs) primarily advising self-management [22]. In October 2020, the NHS announced “post-COVID assessment clinics” [22]. The latest data indicate there are 90 specialist clinics in England [23]. There appear to be no clinics currently in Scotland or Wales [24-26]. Northern Ireland only announced specialist clinics in November 2021 [27]. Patient experience reflects these gaps, exacerbated by HCP dismissal and geographical variations in care (“the postcode lottery”) [9,12,28-30]. In June 2021, the NHS published “Long COVID: The NHS Plan for 2021/22” [22]. However, the real-world rollout and impacts of this plan are not yet known.

### Role of Peer Support

Given care gaps, many long COVID online peer support groups emerged on social media [31]. Peer-led interventions are multifaceted and often used for the management and prevention of various conditions, including HIV/AIDS, diabetes, and adjustment to chronic illness [32,33]. Increasingly, individuals seek medical advice and support online [21,34,35]. In 2012, Ziebland and Wyke identified 7 ways online sources impact patient experiences positively and negatively, including around information, support, health care usage, and creating illness narratives [35]. Although the online landscape has changed substantially due to social media’s rapid recent growth since Ziebland and Wyke conducted their research, it provides strong foundations for this study.

### Importance of This Study

Long COVID research remains sparse [12], focusing on symptomology and social impacts. However, research into support strategies’ impacts is lacking. This study aims to address these epistemological gaps. There is temporal importance: as more people get COVID-19, more will experience long COVID. Cumulative burdens on health care accentuates the need to explore support possibilities.

This study’s aim is to explore the role of online peer support groups in UK adults’ recovery from long COVID, focusing on 2 objectives. The primary objective is to explore the impact these groups have on patients and their experiences within these spaces. The secondary objective is to identify ways these online peer support groups can be situated within broader long COVID recovery planning.

## Methods

### Study Design

I chose qualitative methods to generate richer data, unpacking participants’ experience [36]. Semistructured interviews afforded flexibility; participant responses could shape the interview trajectory [36], while permitting a topic guide (Multimedia Appendix 1). I utilized a phenomenological approach in order to prioritize elucidation of participants’ lived experiences and unpacking of complexities [37,38].

I aimed to recruit 10-12 participants. The decision surrounding sample size was the result of practical constraints, including a condensed time frame for my master’s thesis schedule and burdens placed on me as a researcher with long COVID conducting this research individually. I contacted administrators from 1 Slack and 7 Facebook groups containing over 7500 members for permission to post my recruitment poster, ensuring the post’s appropriateness [39]. Of those, 3 (43%) Facebook groups and the Slack group allowed me to post my recruitment poster. I also shared the poster on my social media to increase exposure.

I used convenience sampling, selecting participants on a first-come, first-served basis. I successfully recruited 11 participants before closing recruitment on July 19, 2021. I only included UK-based adults. Adults have greater control over health-related decisions, and it allowed for focused discussion within the UK health care context.

### Data Collection

I conducted 11 interviews between July 14 and 27, 2021, lasting 26-78 minutes (averaging 49.5 minutes). All participants were offered 2 interviews to ensure they felt their narrative was fully heard, although all opted for 1. I conducted interviews using Zoom, a practical alternative for remote data collection [40,41]. Although initially chosen due to the pandemic, Zoom interviews provided notable advantages. Severe long COVID may preclude travel, reducing participant diversity [41]. It also allowed me to reach people beyond London where support may be less concentrated. Additionally, videoconferencing facilitated more rapport compared to telephone interviews [41]. Only 1 participant kept their video off; however, I kept mine on so they could see my reactions.

I recorded the interviews through Zoom, manually transcribing them on Microsoft Word aided by Express Scribe and an external foot pedal. I transcribed verbatim, reproducing how words were spoken using Poland’s abbreviations [42]. This notation style includes the following: short pauses indicated with dots in parentheses, for example, “(.)” or “(..)”; relaying speech from others and sharing the internal narrative indicated by “(mimicking voice)”; and overlapping speech indicated by a hyphen when the interjection occurs, and the dialogue of the second speaker begins with “(overlapping)” [42]. I cross-checked transcripts against original recordings to ensure validity. I chose to not amend the grammar in participants’ speech in either the transcripts or the writeup. I aimed to capture how words were spoken without “cleaning” the speech with my own biased, uniquely molded, conversational, and linguistic paradigm. To aid clarity in writeup, I used “[…]” to indicate omitted dialogue.

### Data Analysis

After data collection, I utilized reflexive thematic analysis to deeply explore experiences within long COVID online peer support groups that have not yet been formally brought to light. I approached analysis with a constructivist epistemology, stipulating that knowledge is generated through social constructions and variable interpretations [43].

Using Braun and Clarke’s guidance [44], I conducted thematic analysis manually. I coded each transcript inductively—data driven, not using predetermined code lists [36,44]. From my initial code list, I formed related categories using Microsoft Excel. These categories were then grouped into potential themes and subthemes. I refined potential themes, identifying those related specifically to this research [44]. Using Excel, I organized all coded data extracts into each subtheme. I saved documents to provide a comprehensive audit trail of my decision-making throughout the coding process.

Additionally, as I was the sole researcher and interpreter of the data, I engaged in peer debriefing to improve this study’s credibility [45,46]. Peer debriefing is the “process of exposing oneself to a disinterested peer in a manner paralleling an analytic session and for the purpose of exploring aspects of inquiry that might otherwise remain only implicit within the inquirer’s mind” [45]. I chose a peer for this process who is a former colleague with experience in qualitative public health research and with whom I have a relationship built on honesty and trust [47]. We engaged in this process in the final stages of data analysis and reporting to provide additional perspectives on my codes, increase my awareness of any oversights or biases entering the analysis, and troubleshoot redefining my themes.

### Ethical Considerations

The London School of Hygiene & Tropical Medicine’s MSc Research Ethics Committee granted ethics approval for this study (reference numbers 25478, 25478-1, and 25478-2).

The study conformed to the ethical principles of the Declaration of Helsinki. Participants received an information sheet (Multimedia Appendix 2) containing comprehensive study information and my contact details. Following sufficient time for questions or clarification, I obtained informed consent from all participants. Forms, recordings, and transcripts were stored securely. Additionally, I used pseudonyms to protect each individual’s identity owing to the small number of participants in the study.

All efforts were made to ensure interviews were private, though this could not be guaranteed with participants joining Zoom calls from their chosen locations (often, their homes). I was in a private room with headphones and began interviews by enquiring about the risk of their privacy being compromised. If there was a risk, I planned to collaboratively create a code word [48]. Use of this word would have facilitated a conversational shift and prompt friendly close.

Moreover, as participants in my study all experienced long COVID, some may have found interviews exhausting. I reassured them they could manage symptoms, as needed, including ending interviews early. Additionally, interviews could have emotional impacts [36,49], particularly if participants struggled to obtain support in their long COVID journey. I sent participants a document for additional support and guidance, using resources available at the time (Multimedia Appendix 3). Lastly, I checked in with participants to ensure they were not feeling distressed as we concluded the interviews [49].

### Reflexivity and Positionality

As a young adult woman with preexisting health conditions and long COVID following COVID-19 in March 2020, I embodied both researcher and patient. My positionality uniquely impacted the coproduction of knowledge [50,51], providing valuable depth. As an insider, I had the benefit of acute awareness of the issue and understanding language used by participants in describing their experience [52]. Of course, there was a risk of my own biases in analysis and interpretation, such as overseeing data that I take for granted [53,54] or inadvertently allowing my experiences to influence my approach [54]. I avoided these issues by ensuring I considered possible biases in advance and was self-reflexive throughout. Additionally, in engaging in peer debriefing with someone who did not have long COVID, the risk of any subconscious biases influencing data analysis was reduced [45].

I embodied my illness by sitting on the floor during interviews and disclosing it at multiple times. This openness strengthened my connection with participants and broke down research’s traditional unequal power dynamics [51,55]. In being open, participants could ask about my long COVID journey [55]. These questions allowed for a more natural conversational environment, though they shifted conversation off track at times. Additionally, I found myself internalizing the intense emotions [55], particularly when a participant’s journey mirrored my own. I had to create a space where I could step back after interviews to protect my well-being.

## Results

### Summary

**Figure 1:**
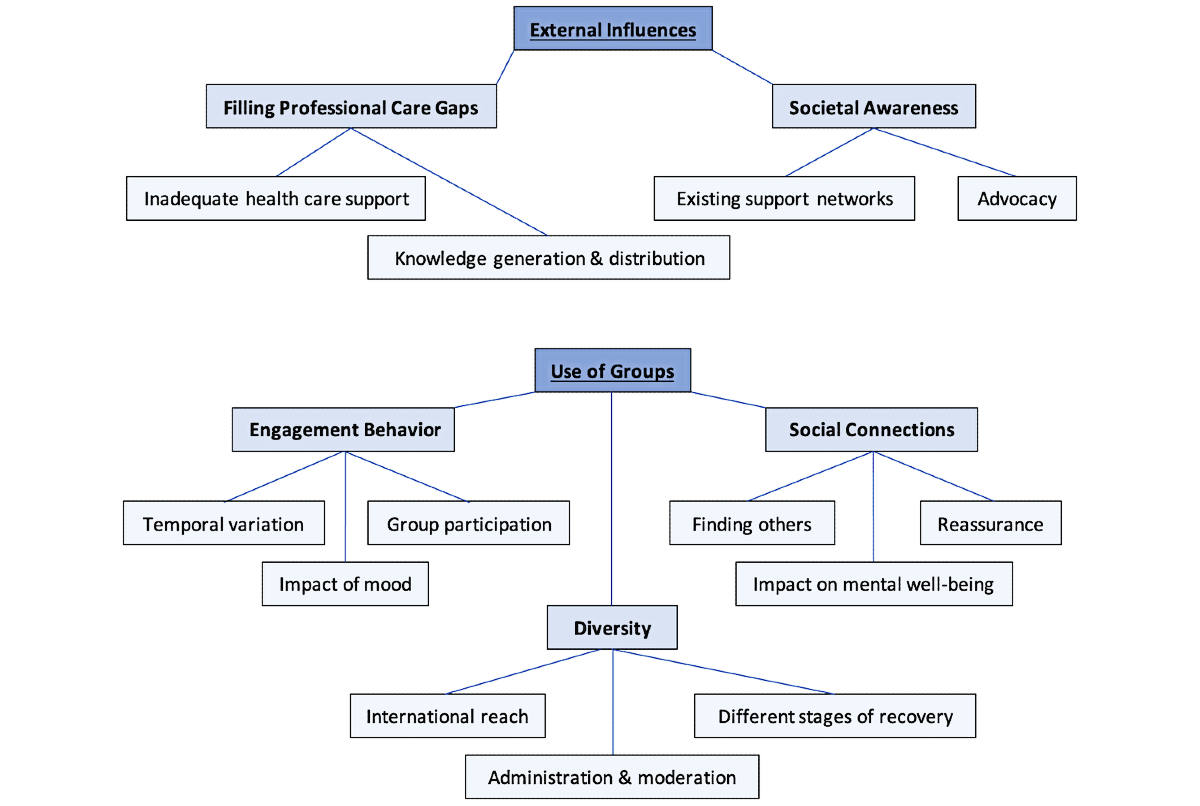
Conceptual thematic map. The hierarchy of concepts identified in thematic analysis are represented here: overarching concepts (bolded and underlined text boxes), themes (bolded text boxes), and subthemes (plain text boxes).

From the interviews, I identified 5 themes and 13 subthemes (see Figure 1). Participants, whose characteristics are described in Table 1, had varying long COVID experiences. In total, 10 (91%) of 11 participants used Facebook for their online support (each with over 7500 members); Jessica used a small WhatsApp group (of roughly 22 group members).

The analysis revealed overarching commonalities between participants’ reflections of these long COVID online peer support groups, which was well articulated by James: “[…] it’s something that’s there, but it’s not what’s needed.” The intricacies of their experience within these groups and the roles groups take on are unpacked in the upcoming themes.

**Table 1:** Participant characteristics (N=11).

Participant (pseudonym)	Age group (years)	Participant-identified gender	Date of (suspected) COVID-19	When they joined the support group(s) (approx)	Support group membership (approx), n
Chloe	30-39	Woman	September 2020	October 2020	2
James	30-39	Man	June 2020	January 2021	1
Jessica	40-49	Woman	May 2020	August 2020	1
Claire	50-59	Woman	February 2021	March 2021	3
Emma	30-39	Woman	October 2020	January-March 2021	3
Oliver	30-39	Man	March 2020	February-March 2021	3-4
Mia	30-39	Woman	January 2021	April 2021	3
Emily	20-29	Woman	January 2021	April 2021	3
Natalie	30-39	Woman	March 2020	June 2021	1
Will	50-59	Man	February 2021	March 2021	1
Sophia	30-39	Woman	October 2020	March 2021	1

### Theme 1: Filling Professional Care Gaps

#### Inadequate Health Care Support

Central to many participants’ narratives was how they turned to online peer support groups after facing an “abyss of silence” [Natalie] from HCPs, feeling let down by health systems.

[…] she said (mimicking voice) I think you probably do have long COVID. And that was like full stop. I kind of paused waiting for some - there was nothing.

Natalie

Although there are (limited) NHS long COVID clinics, participants reported challenges accessing them. For some, there were no clinics nearby that HCPs could refer them to; for others, access remained fraught with complexity.

I’ve been refused access for being too unwell (.) for the COVID clinic, that they say I need a (.) respiratory referral. But I was refused a respiratory referral because I was too well. […] I’m just sat in the middle.

James

The oversight on long COVID care led participants to compare to services offered for other conditions. James noted how with HIV, “at the end of the day there is always somewhere for someone to turn.” Natalie further critiqued the lapses in care more broadly, noting that “often charities and support groups end up filling all the gaps.”

HCPs (gatekeepers to further care) frequently dismissed concerns as mental health issues; patients were “getting gaslighted for that” [Oliver]. Others reported how HCPs repeatedly overlooked otherwise concerning symptoms, opting not to investigate the cause and instead stating, “(mimicking voice) it’s long COVID you just need to (.) um recover” [Chloe]. This lack of adequate attention frustrated participants who were forced to take control over their recovery.

On the groups, some participants wanted more HCP involvement for moderation and “to like answer questions” [Emily]. However, others were content with limited HCP interference, being worried that medical presence “would take away from what the group actually is, which is peer support, isn’t it?” [Mia]. Jessica’s experience on the small WhatsApp group reflected a similar sentiment to Mia’s:

Researcher: And are these doctors and medical professionals - are they part of the group at all? Or is the group just the patients recovering?

Jessica: Um (.) so originally they’re the ones that started it off originally. And then once everybody was sort of introduced, they left and you know - it was so that we could talk about things that were worrying us, without somebody from the medical field […].

#### Knowledge Generation and Distribution

Owing to insufficient medical care, online peer support groups became spaces to share therapies and management techniques. Both Oliver and Natalie used similar language, highlighting the gaps groups aimed to fill:

At the moment we’re all kind of swinging in the dark and kind of hoping to find something […].

Oliver

Mia noted how what was shared in these groups was “actually ahead of the medical information,” coming from patient expertise. Many participants reported gratitude there was advice available, primarily beneficial when no alternatives existed.

However, some were concerned over the lack of content control, meaning they “just take everything with a pinch of salt” [Oliver]. There was palpable frustration regarding potential implications of unregulated content, particularly on social media where “it can be hard to-to differentiate between what is a sensible piece or post and what is a post that’s maybe got ulterior motives to it” [Will].

[…] people might be trying to be helpful, but I think it’s dangerous to try and be too helpful. Because you […] don’t know what their symptoms are, you don’t know what their situation is, you shouldn’t be saying take this take that, because (.) someone could do something stupid and kill themselves.

Will

To avoid risks, several participants used information to signpost HCP discussions, with HCPs as “their safety net” [Sophia]. Although Mia noted risks of overprioritizing medical knowledge:

But then again, (sigh) because there’s a lack of evidence base, there’s an argument there for who is the health professional then?

Mia

This contentious relationship between experts and participants in these groups was tangible.

### Theme 2: Societal Awareness

#### Existing Support Networks

It was clear that discussing long COVID with others was challenging. A few participants expressed fears of burdening loved ones.

[…] my daughter would go (mimicking voice) you alright? And I’d go nope! And I’d just start crying. And that’s when I thought (.) I can’t keep putting everything on her […].

Claire

Others reported feeling friends were not understanding, tiring of the topic, or underplaying the issues. For Sophia, leaving the house to vote was viewed differently by those without long COVID:

That felt like maybe the biggest thing I’d done in a week or a month or something. But (.) to my friend group that aren’t (.) like (.) COVID sufferers or aren’t really aware of how bad it is, they sort of read that and go (mimicking voice) really? That’s an achievement?

Numerous participants reported that support from others with long COVID provided different, more attuned, support:

[…] when it comes from people who have gone through the same thing, it feels a little bit more as in it’s realistic and it will happen.

Jessica

As a result, these peer support groups provided notable advantages to participants’ existing support networks.

Importantly, online support groups also provided spaces for loved ones to better understand long COVID from patients’ perspectives. James’ wife used the space “because I think she was getting a bit frustrated in that (.) (deep inhale) that there is nothing out there that you can just kind of, pick up and read about” [James].

#### Advocacy

Simply the existence of groups validated the condition’s importance. Chloe stated:

I think the groups bringing it to light, that there is so many thousand people feeling this way means that someone’s gonna have to step up and do something.

Additionally, groups facilitated petition sharing and encouraged research, which was deeply valued by participants.

[…] thank God we’ve got people, you know, fighting for us and pushing this research forwards. Because it is an important topic […].

Mia

However, participants felt that groups had limited scope for impact due to societal power dynamics:

[…] on the Facebook groups it is literally Joe Bloggs, it’s no (.) people that have power to change things?

Oliver

When considering where power truly lies, some participants wished for greater government action to provide adequate services, including to have a hand in support groups.

I almost think the Department of Health needs to take a little bit of (..) um control, […] things like the COVID clinics was just - they were completely underfunded before they even start. […] Um (…) we need some sort of, I don’t know, register is probably not the right thing, but some way of identifying people - um (.) traffic-lighting them into support groups. The right support groups for the right people.

James

### Theme 3: Engagement Behavior

#### Temporal Variation

Engagement in the online peer support groups was fluid. Participants joined groups at different times, elucidating different expectations of perceived benefit.

[…] it’s sometimes too soon to be reaching out because I know I didn’t do anything on the group until probably Christmas time. Because I just wanted to see how I would go. […] Whereas I think if you’re too invested (..) you’re never gonna feel like yourself again if you’re constantly reading everyone else feeling miserable.

Chloe

For some, engagement changed over time: “[…] it’s tapered off” [Jessica], they “dip into every now and then” [Will], or, like Oliver, they engaged differently after accessing long COVID clinics.

#### Group Participation

Participants engaged depending on their individual journeys, particularly if they felt their “experiences might be relevant to the question they’ve asked” [Will] or if they “can give some value” to questions asked [Chloe]. Indeed, there was a clear desire to give back to others, either through applying their professional expertise or in offering support.

[…] if I read it and I’ve taken the time to read it, and it’s something that’s resonated with me, I’ll always try and comment back to them and just give them back a little bit of the support that I’ve found from posting on there.

Sophia

#### Impact of Mood

Notably, engagement behavior depended on mood and varied immensely between participants. Some participants used these groups when feeling low:

Because (.) at that point I think you need a wee boost.

Chloe

However, others actively avoided seeing group content in these moments:

[…] on a day when I’m not feeling great I probably avoid looking at it? Um, as much as possible.

Natalie

This discrepancy created an interesting tension, further highlighting how participants held different expectations of the groups’ utility.

### Theme 4: Diversity

#### International Reach

Participants in Facebook groups all noted the international catchment. Several participants highlighted this diversity’s benefits:

I thought it’d be good to hear from other medical systems as well, maybe they’ve got other ideas or different ways of tackling this.

Natalie

However, others expressed potential pitfalls of this global reach, including different terminology causing confusion, overwhelming information, and confronting more stories of suffering. Mia expressed this commonly held sentiment:

[…] sometimes when you’re enlightened to other people’s struggles in other countries, when you’re unwell yourself mentally, it can have quite a big impact I think.

#### Different Stages of Recovery

Groups contained people at all stages of long COVID, with widely varying symptoms and experiences. Many participants compared their experience to others, eliciting a spectrum of emotions. When comparing durations of their illness, there was palpable anxiety and even hopelessness for potential prolonged suffering from reading others’ tumultuous journeys.

[…] people aren’t being negative, and they’re just saying how long their journey is, that to me I think, well I don’t think I can do this for another like (.) 6 months.

Emma

Sometimes it worries me a bit because there’s people on there who’ve had these symptoms for 18 months, and I’m thinking oh my God, please no.

Mia

Others expressed frustration when group members complained about comparatively shorter long COVID journeys.

[…] when you get people that have, that have lost their taste for 4 or 5 weeks and really, really moaning, and I think (.) I know it’s horrible, but some days I think, like, I think some people are a lot further down the line than that.

Emma

Conversely, several participants recognized the benefits of success stories in the groups in providing “hope that there’s light at the end of the tunnel” [Emily]. However, those who recovered often left the online peer support groups. Participants proposed possible reasoning, speculating that “it could trigger some like feelings and memories” [Oliver]. Therefore, success stories were not considered heavily prevalent.

Indeed, when discussions arose surrounding comparing severity of suffering against others in the groups, James noted he found it challenging “just reading the posts because it-it brings up memories of kind of when I was (.) more unwell.” Additionally, some participants reported that seeing reports of worse suffering elicited specific feelings:

[…] a part of me feels a little bit guilty when I read that, and I think oh (chuckles), why am I moaning about my kind of, minor symptoms.

Natalie

Therefore, where participants were in their recovery journeys impacted their experience in the groups.

#### Administration and Moderation

Having peer support groups on relatively open platforms, such as Facebook, unsurprisingly led to discussion around access and moderation. Many groups utilized gatekeeping questions to allow access. Some participants valued this relative privacy. However, James, Oliver, and Mia all expressed concerns; people could simply lie to get into groups. Therefore, this added (albeit imperfect) security held an important purpose in safeguarding users who reasonably believed they were sharing concerns among peers rather than the general public.

Additionally, immense burdens were placed on administrators and moderators for these support groups:

Especially if it’s just like 1 person that’s just decided to make a group that’s all of a sudden got 5000 people with 2000 posts a day.

James

Sophia believed responsibility ought to be shared across group members, as “the admins are also people suffering with long COVID,” to avoid overburdening these few individuals.

### Theme 5: Social Connections

#### Finding Others

Every participant reported joining groups to feel less alone, with several stating, “I was the only kind of person that I knew around me who’d had COVID” [Emily]. There was appreciation for social media’s ability to facilitate connection:

[…] thank God we’ve got all of this online stuff - […] Because if this paned-pandemic had happened when I was a child or whatever, we wouldn’t have any of it!

Claire

However, James’ experience provided an intriguing caveat to the expectation that these platforms can inherently improve connectedness:

Just feeling pretty isolated with it all, and okay, there are other people going through this, but (..) (sigh) That - again it’s (.) that anonymity of different people it (.) it doesn’t really feel - although there is a-a (.) some camaraderie in there, it does kind of almost have that negative impact of feeling kind of more alone (chuckles), conversely.

Indeed, this increased connection with strangers on the internet was not always perceived as a benefit but rather a notable risk.

[…] people are able to get so much detail about you as a person, so sometimes, that does stop me on commenting on things […].

Mia

[…] if someone wants to talk to me about COVID, they can talk to me about it on the group […] I would prefer to do that in an open forum, where there are admins and if, you know, someone does step over the line, I can sort of say, whoa, too far. So I just - I just delete any private request messages that come through. Um, but I think […] there are a lot of vulnerable people in those groups that (.) that may not do that, and therefore then could become a target of various different things.

Sophia

Numerous participants wished for different support group structures to better and more naturally facilitate social connection. Many wanted in-person support, saying that “it would be nice just to sit in a room with 3 or 4 other people and chat through stuff” [James], and Emily noted that this structure could facilitate socializing after periods of isolation.

In the absence of in-person options, either owing to COVID-19 risk or geographical variation among those needing support, several participants noted potential benefits of smaller Zoom calls:

[…] it’s nicer to interact with someone with say face to face than it is over a keyboard, isn’t it?

Will

Importantly, some participants wanted smaller groups (either in their current format or as face to face). However, Sophia did not want to lose the unique benefits that larger and more diverse groups provide:

[…] some of the articles that I’ve found most useful are articles that people have posted because they’ve got completely different issues to me.

#### Reassurance

In finding others, participants felt validated and reassured that they were not alone. Several participants used similar language reflecting fears that their symptoms may be psychosomatic. These groups provided spaces to reassure them that these fears were unfounded.

[…] it was nice to know that people were going through exactly the same thing, that it was wasn’t (…) almost (.) in my head.

Jessica

But when you see huge volumes of other people reporting the same kind of symptoms, […] it reinforces, and you think well actually, this isn’t something that I just made up, […] I’m not being a hypochondriac or, losing my mind.

Natalie

#### Impact on Mental Well-Being

Overall, participants felt these groups helped support their mental well-being in the absence of other care: as encouragement, validation, or an outlet. Chloe’s description of the groups was particularly salient:

[…] these have been a lifeline for so many people, because (.) when the medical services were failing, this was a beacon of light for people.

Participants reported that groups provided support and “would lift” them [Chloe], and they were spaces for unpacking challenging emotions among peers who could better understand.

I just had to turn down my dream job because I’m not well enough to do it. And I just (.)- I was at the bottom of a pit. […] And I just needed to wallow. And that sort of - it gave me somewhere that I could wallow, even though I always try to be positive […]- I just need a few days to wallow in self-pity, and then I’ll - then I’ll be alright again.

Sophia

However, for many participants, these groups negatively impacted their sense of mental well-being and forced them to navigate the groups with particular attention to the emotional toll.

[…] sometimes it can be a little bit overwhelming. I feel sometimes a little bit bombarded with the amount of, sort of, everyone sharing their, sort of, outpourings, of you know. I’ve literally had people saying they feel suicidal, and - […] You know, that’s quite tough to-to read.

Natalie

Sounds a bit selfish really, but you know, it’s nice to check in when you need it really, and to contribute when you need to. Um and then step away from.

Mia

Evidently, these peer support groups imperfectly filled gaps out of necessity. Natalie posed an insightful question on concerns around mental health provision more broadly:

[…] is that filling a void where there should be more mental health support being offered? And, as we know, particularly in this country, there is no mental health support on the NHS, it’s pretty much nonexistent.

Natalie

Similarly, several participants expressed how peer support had been, or they wish it had been, in past experiences to help improve their mental well-being. Natalie shared how she was left searching for support after a terrorist attack:

I knew other people were struggling and other people were then being impacted. […] And I think at times it would’ve been really nice to have been able to talk about the experience with them? And kind of, share it, and then - obviously not to dwell on it too much, but just share it, kind of deal with it, and then, be able to move on […].

When utilized appropriately, peer support could provide immense value in helping people heal and move forward, while supporting their mental well-being.

## Discussion

### Filling the Gaps: What Led People to Groups?

The results evidence how online peer support groups took on vast roles, exemplified in Figure 1, significantly impacting users. Largely, why participants turned to these groups reflected the existing literature, particularly for contested conditions where dismissal is common [9,56]. Participants focused on how online peer support groups filled health care gaps, frequently reporting how HCPs dismissed concerns and disregarded long COVID lived realities. These groups then allowed for condition validation and formed a sense of solidarity against a medical field that is providing insufficient support, as Barker identified with online groups for fibromyalgia [21].

Furthermore, participants’ attempts to seek support reflected experiences by patients with long COVID outside this study, often facing this “postcode lottery” [9,30]. Even when those with long COVID received care, many encountered insufficient compassion or low quality of care [9,12]. James’ experience of being “sat in the middle” is all too common. Participants were left with little choice but to seek out support groups, stumbling upon those on Facebook. Equally, participants reported that they struggled to talk about long COVID with loved ones who could (or would) not understand: a common issue with contested conditions [57-59]. As Allen et al [58] noted with online support for long-term conditions, participants here joined these groups to directly compensate for various unmet needs, whether they be medical, informational, or emotional. The unprompted language of feeling “in the dark” that Natalie and Oliver both used was particularly noteworthy, emphasizing these vast knowledge gaps that online peer support groups had to fill.

### Complexity and Tensions: Unpacking Varying Experiences

Participants used online peer support groups for social connection through shared experience, as in the literature [35,60]. A notable dichotomy was some used groups if they were feeling low, while others avoided them in these moments. Deciding when to engage elucidates participants’ different expectations of perceived benefit. Similarly, engagement was fluid: Participants felt fewer benefits over time, their access to external support changed (for better or worse), or their symptoms fluctuated. Therefore, the flexibility afforded by online peer support groups was distinctly advantageous in providing control [35,61]. Additionally, participants felt empowered when they could support others, as upheld by the literature [62,63]. Of course, individuals in this scenario could face undue burdens [62], though participants did not indicate this, perhaps as responsibility was shared across many group members.

Online support groups provided reassurance. Many participants, like Jessica and Natalie, feared symptoms were all in their head, and thus searched for validation in online peer support groups. Similar to Ziebland and Wyke [35], participants felt a sense of support in these groups, particularly in proving they were not the only ones experiencing this challenging and complex constellation of symptoms. Sharing similar symptomology allowed participants to make sense of their experiences [9,35,59]. Patients coming together around shared illnesses reflects Rabinow’s concept of “biosociality” [64] heavily influenced by Foucault’s “biopolitics,” where bodies are governed through quantification and management [65]. In sharing experiences and knowledge, these groups created “biosocial communities” [66]. Within these communities, “identity work” can occur [59]: members discuss and navigate complex interplays between their condition, society, and self, which brings profound psychological benefit [59]. Unsurprisingly, peer support groups provided space for emotional support [32,35,56,59,67]. Chloe noted how even just having people write supportive comments profoundly benefited her emotional well-being.

However, in addition to emotional support, participants reported emotional impacts driven by the diversity of long COVID experiences encapsulated in groups, including hope, fear, guilt, and frustration. Participants’ experiences reflected Mazanderani et al’s [68] concept of being “differently the same” to negotiate shared illness experience. Participants described their relationship to others in online peer support groups as both beneficial and harmful, reflecting Festinger’s “social comparison theory” in setting up distinctions against others [69]. In this theory, Festinger [69] noted that people tend to use comparisons to others as a means of better conceptualizing their own situations. In the context of these peer support groups, participants engaged in these social comparisons in various ways. Several participants expressed hope when seeing success stories, an example of upward comparison that helped them visualize the possibility of improvement and thus potentially increased their desire to improve themselves to reach that same goal [70,71]. However, others noted the limited positive outcomes shared in groups, potentially causing fear of sufferings’ inevitability and undue stress [72]. Notably, however, upward comparison could cause harm, leading to feelings of jealously and negatively impact self-esteem [71,73,74]. Here, participants were frustrated when seeing those with fewer or less severe symptoms express narratives of deep despondence. Although participants noted users were permitted to use groups in this manner, it dismissed varying experiences. Furthermore, there was evidence of downward comparison in these groups, when individuals look to those who are worse off [75]. This form of comparison caused participants to often feel fear of prolonged suffering, particularly when confronted with narratives from people who experienced long COVID for materially longer than themselves. Interestingly, participants also engaged in downward comparison when they perceived their symptoms to be less “severe” than those of others. This comparison elicited feelings of guilt, though it also created a more implicit sense of gratitude that their experience was not as severe as it could have been.

In contrast to setting situations apart from others, Shiner [76] notes that a central benefit of peer support is the relationality of the peer, particularly in regard to their life experiences. However, with this similarity that emerges inherently in support groups based on particular conditions, there is a notable risk of overidentifying with other’s experiences. Ziebland and Wyke [35] identified this risk when investigating the impact of sharing illness experience online. Here, Chloe similarly voiced concerns of people joining online peer support groups too early in their journeys, thus becoming trapped by their suffering.

### Who Is the Expert? Examining the Patient-Expert Relationship

Participants felt forced into taking control over their recovery rather than relying on “experts.” In doing so, they reflected Rose and Novas’ [66] use of “biological citizenship.” Individuals are expected to take greater responsibility over their health and greater self-management [66]. Participants reported attempts to fill medical gaps through sharing therapies. Some emphasized potential risks of this activity more than others, highlighting varying recovery approaches. Some tried what others with long COVID suggested, as it was the only practical advice available; others were hesitant to try unverified ideas, thus creating a conflict. Interestingly, numerous participants used a similar language to Oliver: they took “everything with a pinch of salt.” This repeated language emphasizes how participants in online peer support groups had to determine safety of suggestions themselves, reflecting burdens placed unduly on patients to critically appraise content. This finding was similar to what Ziebland and Wyke [35] had previously identified; there can be little to distinguish the relative trustworthiness of content that is shared in online spheres.

Patients provided unique forms of knowledge based on embodied illness experience [59,77], often afforded greater credibility by other patients [63]. It is worth invoking Kleinman’s [78] disease versus illness distinction: disease as the biological mechanisms doctors focus on and illness as the “innately human experience of symptoms and suffering.” Long COVID has impacts beyond corporeal disease definitions prioritized by biomedicine; instead, it embodies illness narratives with implications felt heavily at the experiential level. It is therefore unsurprising that these groups attempted to provide both daily functioning and medically oriented support, potentially at a faster rate than would be gained from HCPs [59]. Of course, as many participants noted, there are significant risks with this role: information is no longer authenticated, and significant harm can arise.

Furthermore, participants reported how online peer support groups could be ahead of biomedicine, challenging biomedical and societal assumptions [9,59]. Traditionally, expert authority is not based on patient experiences [35], though participants in these long COVID support groups subverted this narrative. This process is common with contested conditions where patients become “lay experts” [21,59,77,79], unsettling biomedicine’s authority [59,79]. Although professionalization is considered beneficial in empowering patients [59], the results from this study unveil a slightly different story. Of course, having the ability to retain control is essential to avoid becoming entirely disempowered by a dismissive health care system. Nevertheless, embodying expert roles should not necessarily be strived for. Many participants expressed frustration at how people were forced into this role. Despite lay expertise’s benefits, there is continuing conflict between professional and lay experts. Several participants reported taking information from groups to HCPs to guide discussion and advocate for care: a dynamic previously identified in giving patients greater control over care [35,59,80]. Indeed, this behavior could help patients avoid unnecessary tests and appointments [35]. However, HCPs can react negatively if patients’ ideas conflict with their recommendations [81]. Additionally, HCPs remain gatekeepers to care [21]. As a result, interventions available to participants were often limited to over-the-counter medications and at-home exercises.

### Improving the Groups: Participant’s Desires

The literature indicates that online peer support groups offer comparable support to in-person support, with distinct advantages (eg, asynchronous and greater anonymity) [35,61]. Conversely, participants in this study expressed that face-to-face peer support would enhance the experience, facilitating greater connectivity and emotional support without restricted communication “over a keyboard” [Will]. This real-world connection could avoid a potential pitfall of online peer support groups that Ziebland and Wyke [35] identified, where users could get too absorbed in the virtual world at the detriment of their external social worlds. Additionally, participants wished for smaller groups, struggling to feel connected to others in large global groups. Jessica’s overwhelmingly positive experience in her small WhatsApp group could reflect what other participants desired: a closer-knit community with naturally fostered social connection. The craving for more in-person connection may be partially due to reduced face-to-face connection throughout COVID-19 lockdowns; as Emily stated, these groups could aid transitions back into socializing.

Furthermore, the responsibility placed (as participants noted, unduly) on those experiencing long COVID brought up questions about where responsibility ought to lie. With self-management, peers could inappropriately be considered replacements to medical services [32]. Jessica’s experience is an interesting point of comparison. In her case, doctors set up the WhatsApp group and then left, allowing participants to engage without oversight. She appreciated this setup greatly. Those in the Facebook online peer support groups often wished for more professional involvement, either in setup or in content moderation. Having a professional moderator is atypical in online peer support groups, but it can promote engagement [60]. Participants made it clear that there were notable flaws alongside the benefits in the current structure and content of these groups. However, Natalie’s commentary on peer support in other contexts highlights how, if used appropriately, these groups could have immense benefit for those with long COVID.

### Limitations

The relatively small sample size that I used owing to the practical constraints discussed previously had implications on the study. Results may not fully capture the range of experiences that individuals may have in these groups, though it became clear after the first few interviews that consensus was emerging around possible themes. Additionally, the smaller sample size allowed for rich qualitative data generation, providing valuable insights as research begins to explore these online peer support groups in long COVID recovery.

Additionally, selection bias is a risk as individuals volunteered to participate: volunteers’ experiences may not be representative of others. In addition, in sharing my recruitment poster in larger groups to increase outreach, I missed smaller groups. In using a first-come, first-served recruitment methodology due to significant time limitations within my master’s timeline, diversity of participants was limited to those who responded promptly to my recruitment poster. Consequently, generalizability to others in all long COVID online peer support groups is limited.

Furthermore, Zoom interviews had limitations. Individuals with lower technology literacy or limited access to required technology may have been excluded [40]. In addition, 6 interviews experienced technical difficulties. However, I used these technical challenges to build rapport, easing tensions, breaking down power hierarches, and promptly problem-solving together [41].

### Implications

These results fit into the wider discourse, with implications for global public health policy, practice, and research. This study could encourage improvements in the United Kingdom’s long COVID programs to reflect patient needs rather than perceived needs from policy makers and HCPs: embedding appropriate peer support within broader and accessible medically oriented care. Although this study’s UK focus permitted more directed analysis, many groups were international, suggesting possible universality of long COVID care shortfalls. Insights here could aid stakeholders globally in designing, implementing, or participating in long COVID care and peer support specifically. However, this study’s findings may not be entirely transferrable to different health care and sociocultural contexts. For example, in countries with health care systems requiring out-of-pocket payments or complex insurance policies, the reasons to use and the importance of these peer support groups may differ without the same “safety net” of the United Kingdom’s universal health care system. Further research from other countries is essential.

This paper provides an overview of experiences in long COVID online peer support groups. Future research could delve deeper into each subtheme or could explore these groups’ roles and importance among those often marginalized in health care provision (including ethnic minorities, lower socioeconomic groups, and those with disabilities).

### Conclusion

Online peer support groups were *a lifeline but insufficient*. They were imperfect but were needed to fill immense gaps in health care and social support. This study fills epistemological gaps on lived experience of long COVID: beyond corporeal suffering into ways people navigate their newfound reality with a medical field that has yet to catch up. As more attention is given to the condition, hopefully the dark clouds obscuring long COVID will begin to lift, paving the way for more attuned and appropriate care.

## Appendix

Multimedia Appendix 1 Interview topic guide.

Multimedia Appendix 2 Participant information sheet.

Multimedia Appendix 3 Resources for further support.

## Abbreviations

HCP: health care professional
NHS: National Health Service
NICE: National Institute for Health and Care Excellence

## Data Availability

The data generated or analyzed during the study are available from the corresponding author on reasonable request. Available data are in the form of a sufficiently anonymized Microsoft Excel spreadsheet containing all the coded extracts organized into themes and subthemes during thematic analysis. The raw data (transcripts) are not available, as participants provided consent on the basis that only the researcher would have access to these documents.
