# Supplementary material for: Exploring Online Peer Support Groups for Adults Experiencing Long COVID in the United Kingdom: Qualitative Interview Study
Source: J Med Internet Res. 2022 May 20;24(5):e37674. doi: 10.2196/37674 (PMC9128729; doi:10.2196/37674)
Supplement: Multimedia Appendix 1 [file jmir_v24i5e37674_app1.docx]

**Multimedia Appendix 1**. Interview topic guide.

[V1; 13 April 2021]

This is a semi-structured interview, and thus the questions below provide a guide – not a script – in conducting the interviews. Some questions below may be omitted, and others added, depending on where the participant takes the conversation.

**Introduction:**

- Ensure participants are in a comfortable location where they are happy to talk
  - If there is a risk of their privacy being compromised, come up with a ‘code word’ together for them to say or write in the chat
  - Explain what to expect if they use the code word (see end of guide)
- Introduce the project again
- Obtain verbal consent to participate
- Introduce myself and my experience with long-covid
- Set up how they are in control of breaks or ending at any time, stress informal / conversational nature of interview
  - Ensure they feel comfortable expressing their needs to me during the interview
- Ask if they currently are thinking of doing one interview or if they would like to split it into two separate sessions
  - Be clear they can let me know at any point during the interview

**Basic / demographic information:**

- Briefly collect basic demographic information
  - Age, gender
- When they had suspected covid-19 and/or when their long-covid symptoms started

**Why online long-covid support groups:**

- What led you to seek out the long-covid groups?
  - What gap did you hope it would fill?
  - How did you hear about these groups?
  - When in your long-covid journey did you join the group?

**Experience in these groups:**

- What has your experience been in these groups?
  - What do you feel you gain from being part of these groups?
  - How do you use the group? Do you read other posts or ask your own questions / answer others?
  - What have you particularly appreciated about the groups?
  - Is there anything you wish was different in these groups?

*[If participant wants to split the interview into 2 sessions, provide the option to end the first interview here and jump to debrief]*

**Role of shifting patient-expert relationships:**

- What types of interactions and conversations do you see happening?
  - Are participants taking more control over their recovery? In what ways?
  - How is the medical field discussed in these spaces?

**Community-building and advocacy:**

- Have you noticed a sense of community in these spaces?
  - If so, in what ways? Or not?
  - Do you feel connected to the other members of the groups? How?
- How do you think these spaces can contribute to advocacy of long-covid?
  - Have you seen any efforts to improve advocacy outside of those who have long-covid?
  - Is there anything you wish the groups would do to improve advocacy?

**Open question before debriefing**: is there anything else about your experience in the long-covid online support groups that you have not had the chance to share yet?

**Debrief:**

- Check in with how participants feel after the interview
- Provide the additional resources document
- Explain the option of a second interview [if this is their first]
  - Have 3 days from the first interview to email me if they want a second
  - Though no pressure to do so
- Offer email for follow-up contact and for a copy of report when finished

*If participant wants to end interview early due to fatigue or discomfort:*

- Ask them if they want to reschedule to another date
  - Emphasise that there is no pressure to do so – only if they would like to continue sharing their narrative and experience
  - Re-confirm if they are comfortable for me to use the information already collected

*If participant uses their ‘code word’ when privacy is compromised:*

- Immediately switch over to casual conversation for 2 minutes
  - E.g. looking forward to meeting up with family (as though we are friends catching up)
  - Ensure not to arouse suspicion from whoever broke their privacy
- Following ending the call, email participant to check in and offer to reschedule interview if they would like, though it is absolutely their choice
