# Supplementary material for: Exploring Online Peer Support Groups for Adults Experiencing Long COVID in the United Kingdom: Qualitative Interview Study
Source: J Med Internet Res. 2022 May 20;24(5):e37674. doi: 10.2196/37674 (PMC9128729; doi:10.2196/37674)
Supplement: Multimedia Appendix 2 [file jmir_v24i5e37674_app2.docx]

**Multimedia Appendix 2. Participant information sheet.**

**V3; 30 June 2021**

**Title of Project:** Exploring the role and impact of online peer-support groups for adults experiencing long-covid in the UK: a qualitative study

**Introduction:**

I am conducting a research project to explore the role and impact of online peer-support channels for adults experiencing long-covid. I am interviewing a number of individuals and would like to invite you to participate. Before you decide, please read this information sheet carefully, and feel free to ask any questions you may have.

**What is the purpose of the study?**

The purpose of this study is to explore the role of online peer support groups in recovery of adults with long-covid. I will explore what led you to using these groups, and your experience within them, to make recommendations about the role that these groups can play in supporting recovery.

The lived experience and social implications of long-covid remains poorly understood, despite developments in data about the number of people with long-term post-covid symptoms. As healthcare services may get more overwhelmed with the increasing number of people seeking support for long-term post-covid symptoms, these online spaces may become more important as sources of community and validation. This study aims to help inform interventions in long-covid recovery to improve the lived experience of those who have this condition, and better understand whether online support groups are worth promoting.

**Why have I been asked to take part?**

You have been asked to take part because you responded to my recruitment poster. In doing so, you have indicated that you are an adult in the UK experiencing long-covid, and that you are a member of an online support group. I am looking to gather insights into the use of online support groups in recovery journeys.

**Do I have to take part?**

No. You can decide if you would like to take part or not.

**What will happen to me if I take part?**

If you decide to take part, I will ask you to complete the consent form and screening questionnaire. Please allow yourself 4 days from receiving this information sheet to sign the consent form and return it to me, to ensure that you are comfortable with the study and the process. If you have questions, please don’t hesitate to email me.

After I have written consent, we will organise a Zoom interview at a mutually convenient time. You will need a Zoom account. If you do not have one, I am happy to provide instructions for set-up and use if needed. You will have the option of doing one interview or splitting it into two separate sessions. This flexibility will ensure you do not feel pressured to get everything across in one sitting, but also you are not pressured to commit to two sessions.

Interviews will be casual and conversational, lasting approximately an hour – though perhaps shorter or longer depending on how in-depth you are comfortable going. The questions will focus on your experience within these online support groups during your long-covid recovery journey. You do not need to answer any questions you are not comfortable with.

With your permission, the interviews with be audio and video recorded on Zoom. The recordings will be stored securely and only viewed and used by me, the researcher, for the purpose of this research project.

**What are the possible risks and disadvantages?**

If you find talking tiring (on the lungs or from fatigue) as a result of long-covid, you may experience discomfort in the interview. You will have control over when you take breaks, or even end the interview early. You will have the option to reschedule, if you want to, so that you can feel heard without being pushed into discomfort.

Interviews may be long (up to 90 minutes) depending on how much you wish to share. This may cause discomfort from sitting for a long time in front of a screen. You can get up and move around during the interview and look away from the screen. It will be fairly informal, and it is important you are comfortable throughout.

I will do what I can to ensure privacy in the interview. Though you may have a living situation where privacy is hard to have. We will not discuss overly sensitive topics, but I understand that you may be uncomfortable in front of others. Together, we can create a code word that you can use if your privacy has been compromised.

I will be asking questions about sources of support, and it may cause some upset if you have struggled to find support in your long-covid journey. I will provide each participant with resources for seeking further support at the end of the interview.

**What are the possible benefits?**

I cannot promise the study will benefit you directly. However, the information you provide will improve our understanding of the lived experience of long-covid, and the role of online support groups.

You will be contributing to important research on long-covid, an area that has not been sufficiently understood or researched. Your contribution in this project will help us know whether online support groups are a potentially useful option to include in interventions to aid those recovering from long-covid.

**Can I change my mind about taking part?**

Yes. You can withdraw from the study at any time. You just need to inform me of your decision. You will not experience any negative impacts from withdrawing early. I will still provide you with a list of resources, should you find them helpful.

**What will happen to information collected about me?**

I will store your consent form document on an encrypted, password protected external USB drive. The interviews will be recorded, with your consent, and then transcribed. The recordings and transcriptions will be made anonymous using a participant number, and will be stored securely in an encrypted, password protected folder on my laptop. Only I will have access to all this information.

Your name, or other identifiable information, will not be linked to quotations or narratives used in the project. You will be assigned a codename in the write up to differentiate your experience with other respondents. Other than your experience using long-covid support groups, the only information that may be discussed is your age, gender, and how long you have had long-covid symptoms. From this information, you will not be able to be recognised.

**What are your choices about how your information is used?**

You can stop taking part in the study at any time, without giving a reason. You have the choice to either have all your data be destroyed, or you can let me know if you are happy to allow the data already collected to be used in analysis and write up.

You also will have the choice to allow me to use direct quotations from the transcripts, or whether you would prefer that only the general themes and ideas are used from your interview.

**What will happen to the results of this study?**

The results of this study will be written up into a Masters (MSc) thesis project. The findings may be published in journals so that the information can be shared to further inform recovery planning for long-covid. Your personal information will not be included in the project, and there is no way that you can be identified from it.

You are more than welcome to contact me to follow how the research is progressing. When the final research report is available, I will be happy to provide you with a copy if you are interested.

**Who has reviewed this study?**

All research involving human participants is looked at by an independent group of people, called a Research Ethics Committee. This study has been reviewed and given a favourable opinion by The London School of Hygiene and Tropical Medicine Research Ethics Committee.

**Further information and contact details:**

Thank you for taking the time to read this information sheet. If you are happy to take part in the study, please **read and sign the consent form and screening questionnaire**.

If you would like any further information, please contact the me below as I can answer any questions you may have about the study.

Contact details:

[Researcher email address redacted]
