# Supplementary material for: Exploring Online Peer Support Groups for Adults Experiencing Long COVID in the United Kingdom: Qualitative Interview Study
Source: J Med Internet Res. 2022 May 20;24(5):e37674. doi: 10.2196/37674 (PMC9128729; doi:10.2196/37674)
Supplement: Multimedia Appendix 3 [file jmir_v24i5e37674_app3.docx]

**Multimedia Appendix 3. Resources for further support.**

[V1; 10 April 2021]

This document includes information and resources that you may find helpful on both long-covid management and psychological support.

1. **Your COVID Recovery**: an NHS website offering advice about managing long-covid symptoms at various stages of recovery.

*Website*: <https://www.yourcovidrecovery.nhs.uk/>

1. **Long Covid Support**: a peer support and advocacy group for individuals with long-covid. The webpages contain information about symptom management, and resources including GP letter templates, support groups, and benefits and finances.

*Website*: <https://www.longcovid.org/>

1. **Post-COVID HUB**: a website created by Asthma UK and British Lung Foundation to offer support on symptom management and resources on recovery.

*Website*: <https://www.post-covid.org.uk/>

*Team of respiratory specialists 9am-5pm (Mon-Fri):* 0300 222 5942

1. **The Samaritans**: for someone to talk to 24/7 without any judgement or pressure. The website also offers information about self-help and other resources if you are having a difficult time at the moment.

*Website*: <https://www.samaritans.org/>

*Call for support (for a one-to-one call immediately)*: 116 123

*Email for support (for a response within 24 hours)*: [jo@samaritans.org](mailto:jo@samaritans.org)

1. **Young Minds**: offers a range of resources about mental health, specific to young people, as well as urgent support if you are experiencing a mental health crisis.

*Website*: <https://youngminds.org.uk/>

*Crisis Messenger*: Text YM to 85258

1. **Mind**: offers resources and advice for anyone with a mental health problem. They have dedicated sections on covid-19, as well as an Infoline to call for information about accessing further support.

*Website*: <https://www.mind.org.uk/>

*Infoline phone number*: 0300 123 3393
